# Pseudotumor cerebri presenting with visual failure in promyelocytic leukemia: a case report

**DOI:** 10.1186/1752-1947-6-408

**Published:** 2012-11-29

**Authors:** Fahid T Rasul, Ahmed K Toma, Akbar A Khan, Gordon T Plant, Laurence D Watkins

**Affiliations:** 1 Victor Horsley Department of Neurosurgery, National Hospital for Neurology and Neurosurgery, Queen Square, London, WC1N 3BG, UK

## Abstract

**Introduction:**

Pseudotumor cerebri secondary to all-trans retinoic acid in acute promyelocytic leukemia is a reported but rare complication of the therapy. Most cases improve following the discontinuation of all-trans retinoic acid. There is no published literature on how to manage such patients if severe symptoms of increased intracranial pressure continue after discontinuation of the drug.

**Case presentation:**

We report the case of a 16-year-old Afro-Caribbean woman with aggressive secondary pseudotumor cerebri who presented to our facility with visual failure that persisted despite discontinuation of all-trans retinoic acid. A lumbar drain was inserted for 11 days resulting in symptomatic relief of headaches and objective improvement of visual failure. Pressure settings were titrated regularly to ensure optimal symptomatic relief.

**Conclusions:**

The use of a lumbar drain for continuous drainage of cerebrospinal fluid in patients with all-trans retinoic acid-induced pseudotumor cerebri resistant to all-trans retinoic acid discontinuation is a feasible management option. This method can be used when other less invasive measures have failed to improve signs and symptoms. Permanent drainage of cerebrospinal fluid with a shunt may also provide a long-term viable management strategy but the use of a lumbar drain may be preferable if the cause of pseudotumor cerebri is known to be self-limiting.

## Introduction

Acute promyelocytic leukemia (APL) is a distinct subset of acute myeloid leukemia characterized by an abnormal fusion protein promyelocytic locus gene (PML)/retinoic acid receptor α (RARA) [1]. All-trans retinoic acid (ATRA) is a common treatment for APL. It degrades the PML/RARA fusion protein causing terminal differentiation of malignant cells in the bone marrow as indicated by the presence of Auer rods [2]. This is followed by the re-emergence of normal hematopoietic cells as patients achieve complete remission. ATRA is associated with a high incidence of complete remission in patients newly diagnosed as having APL [3]. Pseudotumor cerebri is a rare but well described side effect of ATRA therapy in patients with APL, most commonly children [4,5]. It is characterized by headache, nausea and/or vomiting, papilledema and diplopia. We present a case of pseudotumor cerebri that was resistant to normal therapies, which only resolved when a previously unreported intervention (insertion of a lumbar drain) was employed.

## Case presentation

A 16-year-old Afro-Caribbean woman was diagnosed as having APL and given ATRA. Three weeks later she presented to our emergency department with severe headache and visual loss. This was thought to be secondary to ATRA treatment. Serum analysis and diagnostic lumbar puncture (LP) were performed confirming the absence of other causes. ATRA was discontinued. Symptoms of increased intracranial pressure persisted despite discontinuation of the drug.

Intrathecal cytarabine was administered twice per week and a total of five cycles were given over a period of 18 days. The papilledema remained stable during this period. Dexamethasone was also given. Six lumbar punctures were performed over this 18-day period. The opening pressures ranged from 28cmH_2_0 to 43cmH_2_0. Between 30 and 50mL of cerebrospinal fluid (CSF) was drained on each occasion. Unfortunately headaches and visual loss persisted despite these interventions. Computed tomography (CT) imaging of the brain on day 18 showed mildly reduced size ventricles with moderate effacement of cortical subarachnoid spaces. Secondary pseudotumor cerebri was diagnosed and our patient was transferred to a specialized neurosurgical unit for further care.

**Figure 1:**
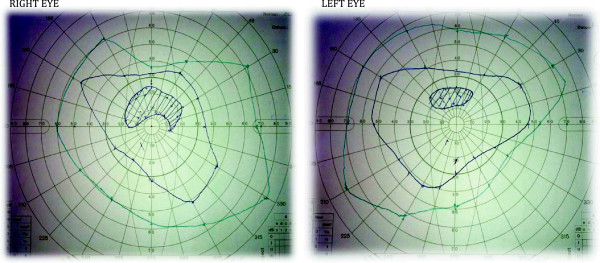
Visual field charts prior to removal of lumbar drain.

A lumbar drain was inserted under sedation at the L3/4 level for continuous drainage of CSF. A 20G needle was used with our patient lying in the fetal position and the bevel facing upwards to ensure accurate measurement of opening and closing pressures. The opening pressure was noted to be 37cmH_2_0. Neuro-ophthalmological assessment confirmed swollen discs consistent with severe papilledema at the time of lumbar drain insertion. Our patient remained in bed rest for 11 days. The pressure setting on the lumbar drain was adjusted according to our patient’s signs and symptoms. Pressure settings ranged from 5cmH_2_0 to 10cmH_2_0 to ensure optimal CSF drainage. Pressures as high as 26cmH_2_0 were observed during the lumbar drain phase. Daily neuro-ophthalmology review showed significant improvement in papilledema over the 11-day period for which the lumbar drain remained *in situ*. On the day of admission to the neurosurgical unit the visual acuity of our patient was recorded as CF1 (counting fingers at 1 foot distance) in the right eye and CF10 in the left eye. On day seven the visual acuity was recorded as 6/60 in the right eye and 6/24 in the left eye. On day 11, prior to removal, visual acuity was 6/18 in the right eye and 6/12 in the left eye. Visual field charts on day 11 are shown in Figure 1. The marked improvement in papilledema and visual acuity over a short period is unusual and is discussed further in the discussion section. Gradual symptomatic improvement occurred over the first seven days. The drain was removed four days later when the pressure had stabilized at 12cmH_2_0 level. After removal of the drain our patient experienced low-pressure headaches for three days. These were self-limiting and had disappeared two days prior to discharge. Our patient suffered no subsequent symptoms and signs of increased intracranial pressure and remained well at three-month follow-up.

## Discussion

Acute promyelocytic leukemia (APL) is a distinct subset of acute myeloid leukemia. It represents five percent of all acute myeloid leukemia’s. It is characterized by abnormal, heavily granulated promyelocytes. The precise pathophysiology is unknown. However it has been speculated that an excess of vitamin A enhances production of CSF and alters the lipid constituents of arachnoid villi causing impaired absorption of CSF [6]. ATRA is commonly used to treat patients with APL. It provides a better complete remission rate compared to chemotherapy alone [3]. Pseudotumor cerebri is a recognized but rare complication in APL. It is characterized by signs and symptoms of increased intracranial pressure, no signs of hydrocephalus on brain imaging and a high opening pressure on lumbar puncture. Early diagnosis is important to ensure appropriate therapy is administered to prevent persistent visual impairment and even blindness [7].

Several cases of pseudotumor cerebri secondary to ATRA therapy have been described. There is conflicting evidence for the need to discontinue ATRA treatment in patients with recognized pseudotumor cerebri secondary to the drug. It has been postulated that the effect of ATRA diminishes with time due to pharmacological adaptation resulting in reduced serum concentration after prolonged treatment [8]. Some reports have suggested that ATRA-induced pseudotumor cerebri is self-limiting while others have suggested improvement only on discontinuation of ATRA [9,10]. In our patient’s case, her symptoms persisted for more than three weeks after discontinuation.

It is likely that the drug can give rise to a spectrum of severity of raised intracranial pressure ranging from a mild self-limiting disorder to one that is severe and persistent, as was the case with our patient, with a real risk of blindness.

Only rarely is there a need for drainage of CSF for symptomatic improvement. To the best of our knowledge, we present the first case in which pseudotumor cerebri persisted despite discontinuation of ATRA and serial therapeutic lumbar punctures.

It has been reported that promyelocytic leukemia is associated with venous sinus thrombosis [11]. Furthermore dural venous sinus thrombosis is a recognized cause of pseudotumor cerebri [12]. It is therefore possible that the observed pseudotumor cerebri was caused by venous sinus thrombosis. A CT venogram is the investigation of choice for the diagnosis of venous sinus thrombosis and allows physicians to start anti-coagulation therapy in the form of a heparin infusion or treatment dose low molecular weight heparin if the investigation is positively diagnostic. In our patient’s case this investigation was not performed, however, we suggest that this could be an additional diagnostic tool that may be utilized giving another alternative treatment if found to be positive for venous sinus thrombosis. There have been some case series suggesting that venous sinus stenting is an effective treatment for pseudotumor cerebri [13]. However, currently it is not a widely used treatment modality in the UK. The time course of improvement observed in our patient is consistent with recanalization of a dural venous sinus after thrombosis. The lumbar drain exerts its effect by reducing intracranial pressure (ICP). Our patient showed significant improvement in their symptoms of raised ICP as we titrated the lumbar drain settings to symptomatic effect. If the primary cause for pseudotumor cerebri was venous sinus thrombosis the treatment with a lumbar drain was useful in improving our patient’s symptoms over the period it took for recanalization of the venous sinus. The time course for recanalization is unpredictable and therefore repeated lumbar punctures is not recommended while awaiting recanalization due to the repeated exposure to the risks and complications of this procedure. This may explain the rapid improvement in papilledema. CSF diversion via a lumbar drain is not known to improve papilledema so rapidly, although optic nerve sheath fenestration has a much more rapid action. It is possible that the lumbar drain allowed recanalization of a venous sinus, which caused a rapid improvement of papilledema. In this way the lumbar drain ‘broke the cycle’ of pseudotumor cerebri.

On insertion of a lumbar drain we were able to titrate the pressures to ensure adequate drainage of CSF to improve our patient’s signs and symptoms. Additional measures that could be used if this approach did not provide a long-term solution would include the insertion of a shunt for permanent CSF diversion. This is commonly used in patients with pseudotumor cerebri of other etiologies. In this situation a CT venogram would be more useful as it may confirm or exclude venous sinus thrombosis as the primary cause. This may affect management as a dural sinus stent may be considered as an alternative, permanent treatment in persistent cases although this procedure is only performed in select centers currently.

## Conclusions

In pseudotumor cerebri secondary to ATRA in patients with APL, placement of a lumbar drain is a viable management strategy until signs and symptoms of increased intracranial pressure improve. It is an invasive low risk surgical procedure. Pressure can be titrated to suit the patient’s signs and symptoms. Other, more long-term interventions may include permanent CSF diversion via a shunt. Careful consideration should be made to investigate the etiology of this rare condition in individuals if this will affect management decisions.

## Consent

Written informed consent was obtained from the patient’s legal guardian for publication of this case report and any accompanying images. A copy of the written consent is available for the Editor-In-Chief of this journal.

## Competing interests

The authors declare that they have no competing interests.

## Authors’ contributions

FTR: data collection and manuscript formation. AKT: surgical intervention, AAK: manuscript advice. GP: Ophthalmology opinion and review. LDW: manuscript advice and lead neurosurgeon in charge of patient care. All authors read and approved the final manuscript.
